# Quantitative assessment of retinal microvascular remodeling in eyes that underwent idiopathic epiretinal membrane surgery

**DOI:** 10.3389/fcell.2023.1164529

**Published:** 2023-04-20

**Authors:** Yingjiao Shen, Xin Ye, Jiwei Tao, Chenhao Zhao, Zhaokai Xu, Jianbo Mao, Yiqi Chen, Lijun Shen

**Affiliations:** ^1^ National Clinical Research Center for Ocular Diseases, Eye Hospital, Wenzhou Medical University, Wenzhou, China; ^2^ Department of Ophthalmology, Zhejiang Provincial People’s Hospital, Hangzhou, China

**Keywords:** idiopathic epiretinal membrane, optical coherence tomography angiography, vessel tortuosity, vitrectomy, microvascular remodeling

## Abstract

**Purpose:** To explore the surgical outcomes of the macular microvasculature and visual function in eyes with idiopathic epiretinal membrane (iERM) using spectral-domain optical coherence tomography angiography (SD-OCTA).

**Methods:** This observational, cross-sectional study included 41 participants who underwent iERM surgery with a 3-month (3M) follow-up. Forty-one healthy eyes formed the control group. The assessments included best-corrected visual acuity (BCVA) and mean sensitivity (MS) by microperimetry and SD-OCTA assessment of vessel tortuosity (VT), vessel density (VD), foveal avascular zone, and retinal thickness (RT).

**Results:** The findings showed statistically significant differences in VT, foveal avascular zone parameters, RT, BCVA, and MS between the iERM and control groups (*p* < 0.05)**.** After iERM surgery, the macular VT, SCP VD, and RT decreased significantly (*p* < 0.01) while the DCP VD increased (*p* = 0.029). The BCVA improved significantly (*p* < 0.001) and was associated with the MS (rs = −0.377, *p* = 0.015). MS was associated with the SCP VD and RT at 3M (SCP VD rs = 0.511, *p* = 0.001; RT rs = 0.456, *p* = 0.003). In the superior quadrant, the MS improved significantly (*p* < 0.001) and the improvement of MS was associated with the reduction of VT (*β* = −0.330, *p* = 0.034).

**Conclusion:** Microcirculatory remodeling and perfusion recovery were observed within 3 months after iERM surgery. VT was a novel index for evaluating the morphology of the retinal microvasculature in eyes with iERM and was associated with MS in the superior quadrant.

## Introduction

Idiopathic epiretinal membrane (iERM) is a common macular disease characterized by abnormal glial proliferation in the vitreoretinal interface. Proliferative cells in the macular area, migrating along the surface of the internal limiting membrane (ILM), can cause wrinkling and retinal traction (Fung et al., 2021). The contraction of the iERM is responsible for the additional thickening, folding, or puckering, along with vascular distortion, and the traction on the retina can alter the morphology of the fovea, leading to clinical symptoms such as metamorphopsia, blurred vision, and decreased visual acuity (Steel and Lotery, 2013). Pars plana vitrectomy, followed by peeling of the membrane and ILM, is the recommended therapy for treating iERM, which can normalize the wrinkled retinal surface and thickened macula (Far et al., 2021).

Optical coherence tomography (OCT) is widely applied in the assessment and diagnosis of macular diseases. However, OCT cannot be used to visualize retinal blood flow (Koustenis et al., 2017). OCT angiography (OCTA), a newly introduced imaging modality based on OCT, is used extensively because of its non-invasiveness in visualizing the retinal vessels. It provides a detailed en-face view of each capillary layer. In addition, the high resolution of the capillary network facilitates better visualization of the retinal vasculature in multiple layers and more reliable assessments of vascular features. Previous reports have described OCTA-derived indexes, including foveal avascular zone (FAZ) parameters, vessel density (VD), and fractal dimension (Kim and Park, 2021; Mao et al., 2021).

Normal retinal blood vessels are straight or slightly curved but may become dilated and tortuous in several conditions, including angiogenesis, high blood flow, and blood vessel congestion (Hart et al., 1999). Therefore, vessel tortuosity (VT) was also a meaningful indicator of microvascular evaluation, which could infer the severity and the progression of many retinopathies. Previous studies evaluated VT in sickle cell retinopathy (SCR), (Alam et al., 2019), diabetic retinopathy (DR) (Alam et al., 2020; Alam et al., 2021), and familial retinal arteriolar tortuosity (FRAT) (Saraf et al., 2019), among others. However, few studies have evaluated VT in eyes with iERM. Additionally, sensitivity based on microperimetry is a good functional parameter, but it is rarely used in research on microperimetry assessment for iERM. In contrast with automated perimetry, it demonstrates better reliability and retest-variability (Pfau et al., 2021). Only a few studies have combined OCTA with microperimetry to evaluate retinal changes in eyes with iERM (Feng et al., 2021; D'Aloisio et al., 2021). Therefore, we aimed to explore the changes in retinal microvascular architecture and visual function in the macular region in iERM patients using OCTA images.

## Materials and methods

We examined 41 eyes of 41 patients affected by iERM that were admitted to the Affiliated Eye Hospital of Wenzhou Medical University between January 2019 and December 2021. All patients underwent 23-gauge pars plana vitrectomy combined with iERM peeling and non-foveal-sparing ILM peeling by a senior surgeon (SLJ). Patients with mild cataract who were older than 55 years old also underwent phacoemulsification and intraocular lens implantation. Matched for age, 41 healthy eyes of 41 healthy participants were used as controls. This study used anonymous retrospective data and did not require active patient participation or informed consent. All procedures adhered to the tenets of the Declaration of Helsinki. The study was reviewed and approved by the Medical Ethics Committee of the Affiliated Eye Hospital of Wenzhou Medical University.

Before and after surgery, all enrolled patients received a comprehensive ophthalmic evaluation, including the assessment of best-corrected visual acuity (BCVA), slit-lamp biomicroscopy, indirect fundus ophthalmoscopy, OCTA, and microperimetry. The criteria for inclusion were: 1) diagnosis of iERM by retinal experts based on the results of fundus examination, OCT, and OCTA; and 2) no previous history of vitreoretinal prior surgery. The criteria for exclusion were: 1) previous history of ocular diseases such as retinal vascular occlusions, retinal detachment, trauma, high myopia, and uveitis; 2) previous history of systemic disorders, including systemic hypertension and diabetes; and 3) poor image quality (signal strength index less than 5) due to poor image fixation or obvious opacity in refracting media, such as severe cataract and leukoplakia.

### Image acquisition

All images were acquired with spectral-domain OCTA (SD-OCTA) (Optovue, Fremont, CA, United States). All the OCTA images had a field of view of 6 mm × 6 mm. Retinal vasculature was assessed within two horizontal retinal slabs of the OCTA, including the superficial capillary plexus (SCP) and deep capillary plexus (DCP), spanning from the ILM to the superficial inner plexiform layer and from the deep inner plexiform layer to the outer plexiform layer, respectively. For each eye, three 6 × 6-mm OCTA volume scans were acquired at baseline, 1 month (1M), and 3 months (3M) after surgery.

### Image processing

The primary indicators included: 1) VT; 2) SCP VD; 3) retinal thickness (RT); 4) DCP VD; 5) FAZ area (FAZa), perimeter (FAZp), and acircularity index (AI). While analyzing the former three parameters, we considered the temporal, superior, nasal, and inferior sectors of a circular zone with a diameter of 6 mm. VD, RT, FAZa, and FAZp were obtained from the built-in image analysis software of the OCTA device. FAZ AI was calculated as the measured FAZp divided by the perimeter of the regular circle with the same FAZa.

**FIGURE 1:**
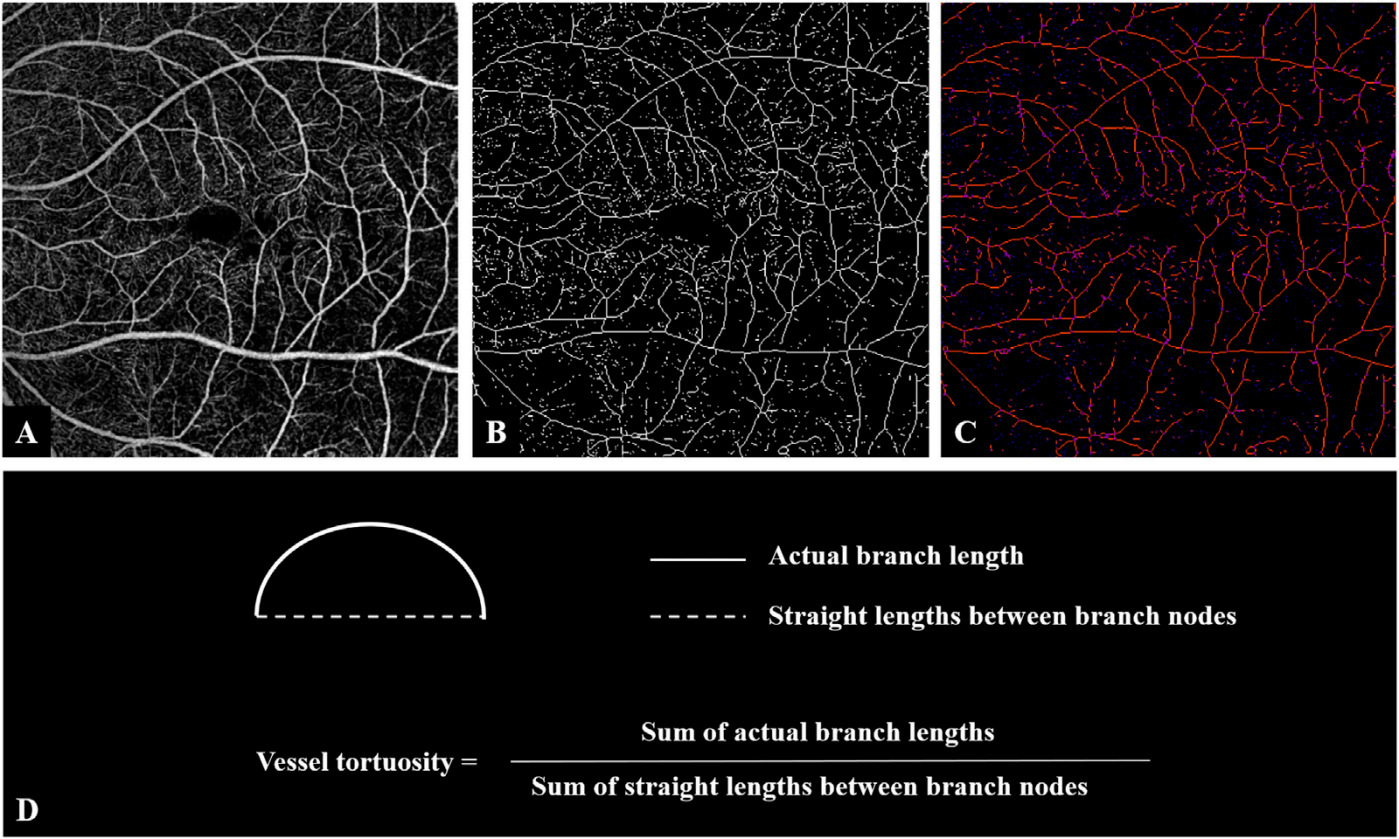
Image processing steps for analyzing vessel tortuosity. **(A)** OCTA image of the superficial retinal layer of an eye with iERM. **(B)** Binarized vessel was skeletonized. **(C)** The vessel branches and the branch nodes were obtained and the lengths of both actual branches and the imaginary straight lines between nodes were generated. **(D)** Vessel tortuosity was calculated as the sum of the branch lengths divided by the sum of the lengths of the imaginary straight lines. Abbreviations: iERM, idiopathic epiretinal membrane; OCTA, optical coherence tomography angiography.

To calculate VT, we exported the OCTA images of SCP (Figure 1A) and used ImageJ for further feature extraction and image analysis. According to the method described previously (Lee et al., 2018), we converted the original images to 8-bit grayscale images and used the “Trainable Weka Segmentation” plugin to binarize the vessels. After image binarization, using the “Skeletonize” plugin and acquiring a thin track of vessels with a 1-pixel diameter (Figure 1B). The “Analyze skeleton” plugin in ImageJ was used for the calculation of the length of vessels, including the actual length of each branch and the imaginary straight length between the two branch nodes (Figure 1C). VT was calculated as shown in Figure 1D. If needed, manual segmentation was performed to assess the entire retina to prevent segmentation errors. Images with poor quality, such as those with poor contrast due to poor image fixation or media opacities, were excluded. When erroneous segmentation results were caused by poor image quality, we discarded the analysis for obvious failure cases.

### Functional assessment

Microperimetry was performed with MP-3 (NIDEK, Gamagori, Japan) at the baseline and 1M and 3M postoperatively. All patients underwent the examination under dim-light conditions. The fixation target of MP-3 was a red ring with a diameter of 1 with a white monochromatic background at 31.4 abs. The fovea was marked by a blue star. An automatic eye tracker was used with a customized grid of 61 points covering the central 20° centered on the fovea (approximately 6 mm × 6 mm range). The retinal sensitivities were approximately 0–36 dB. In this study, the mean sensitivity (MS) of all 61 points and the MS of the four quadrants of the 15 points were calculated. BCVA was performed using a Snellen chart, and trained optometrists recorded measurements before and after the surgery. For statistical analysis, BCVA was converted to a logarithm of the minimal angle of resolution (logMAR).

### Statistical analysis

All statistical analyses were performed using SPSS v26.0 (SPSS for Windows, Chicago, IL, United States). All quantitative variables were described as mean ± standard deviation (SD). A Shapiro–Wilk test was performed to evaluate the departure of each variable from a normal distribution. The independent-sample t-test was performed for the difference in age between the groups. The Chi-squared test was performed for the difference in sex between the groups. The Wilcoxon signed-rank and Kruskal–Wallis H tests were used to compare the parameters. Spearman’s Rho correlation coefficient was used to evaluate the correlations between the anatomical and functional parameters. Multivariate linear regression was used to analyze the correlations between the functional and anatomical variables. *p* < 0.05 was considered statistically significant.

## Results

We included 41 eyes of 41 patients diagnosed with iERM as the iERM group and 41 healthy eyes of 41 persons as the control group. During vitrectomy, 39 of 41 eyes were treated by phacoemulsification for cataracts. One eye was pseudophakic. One eye did not undergo cataract surgery because the patient was relatively young with no apparent cataract. No statistically significant differences were found in age (*p* = 1.000) and sex (*p* = 0.557) between the iERM and control groups (see Table 1).

**TABLE 1:** Comparison of baseline parameters between the iERM (N = 41) and control (N = 41) groups.

Baseline parameter	iERM group	Control group	*p*-value
**Age (year)**	66.20 ± 8.68	66.20 ± 8.74	1.000
**Sex (M/F)**	8/33	6/35	0.557
**Anatomical parameters**			
**VT**	1.1165 ± 0.0226	1.1072 ± 0.0129	**0.047**
**SCP VD (%)**	48.06 ± 5.96	48.51 ± 3.76	0.856
**DCP VD (%)**	45.14 ± 6.43	48.06 ± 5.37	0.078
**FAZa (mm** ^**2**^ **)**	0.07 ± 0.10	0.30 ± 0.11	**< 0.001**
**FAZp (mm)**	1.04 ± 0.53	2.12 ± 0.38	**< 0.001**
**FAZ AI**	1.25 ± 0.20	1.12 ± 0.06	**< 0.001**
**RT (μm)**	359.93 ± 52.58	284.44 ± 15.91	**< 0.001**
**Functional parameters**			
**MS (dB)**	22.27 ± 3.87	26.18 ± 1.93	**< 0.001**
**BCVA (logMAR)**	0.49 ± 0.38	0.01 ± 0.04	**< 0.001**

Bold font indicates statistically significance (*p* < 0.05).

Abbreviations: iERM, idiopathic epiretinal membrane; VT, vessel tortuosity; VD, vessel density; SCP, superficial capillary plexus; DCP, deep capillary plexus; FAZ, foveal avascular zone; FAZa, FAZ area; FAZp, FAZ perimeter; FAZ AI, FAZ acircularity index; RT, retinal thickness; MS, mean sensitivity; BCVA, best-corrected visual acuity; logMAR, logarithm of the minimal angle of resolution.

### Microvascular remodeling

At baseline, the iERM group had a higher VT, smaller FAZa, smaller FAZp, larger FAZ AI, and greater RT than the control group (VT 1.1165 ± 0.0226 vs. 1.1072 ± 0.0129, *p* = 0.047; FAZa 0.07 ± 0.10 mm^2^ vs. 0.30 ± 0.11 mm^2^, *p* < 0.001; FAZp 1.04 ± 0.53 mm vs. 2.12 ± 0.38 mm, *p* < 0.001; FAZ AI 1.25 ± 0.20 vs. 1.12 ± 0.06, *p* < 0.001; RT 359.93 ± 52.58 μm vs. 284.44 ± 15.91 μm, *p* < 0.001). No significant differences were found in the SCP VD and DCP VD (SCP VD 48.06% ± 5.96% vs. 48.51% ± 3.76%, *p* = 0.856; DCP VD 45.14% ± 6.43% vs. 48.06% ± 5.37%, *p* = 0.078) (see Table 1).

**FIGURE 2:**
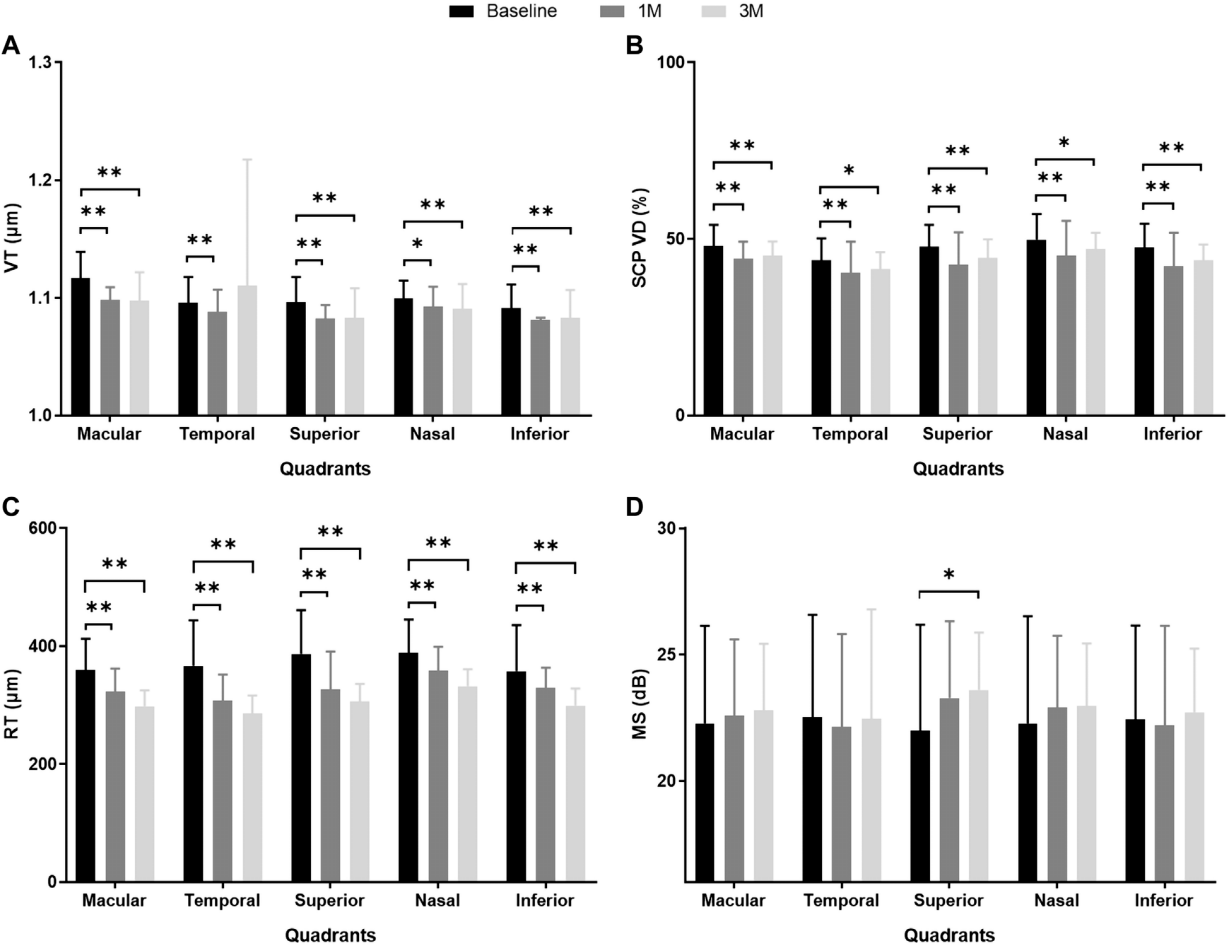
MS and anatomical parameters of the macular region in eyes with iERM observed at baseline and 1 month (1M) and 3 months (3M) after vitreoretinal surgery. **(A)** VT; **(B)** SCP VD (%); **(C)** RT (μm); **(D)** MS (dB). **p < 0.01, *p < 0.05. Abbreviations: iERM, idiopathic epiretinal membrane; VT, vessel tortuosity; SCP, superficial capillary plexus; VD, vessel density; RT, retinal thickness; MS, mean sensitivity.

Compared to the baseline values, there were significant reductions in VT, SCP VD, and RT in the macular region during the 3-month follow-up (VT, from 1.1165 ± 0.0226 to 1.0978 ± 0.0239, *p* < 0.001; SCP VD, from 48.06% ± 5.96% to 45.17% ± 4.11%, *p* = 0.002; RT, from 359.93 ± 52.58 μm to 298.24 ± 26.87 μm, *p* < 0.001). The DCP VD of the macular region increased from 45.14% ± 6.43% to 48.32% ± 4.86% over 3 months (*p* = 0.029). No significant differences were found in the FAZa (*p* = 0.053), FAZp (*p* = 0.140), and FAZ AI (*p* = 0.228) at 3M relative to the baseline (see Table 2; Figure 2).

**TABLE 2:** Comparison of functional and anatomical parameters of the macular region at baseline and 3 months (3M) after surgery in eyes with iERM.

Parameters	Baseline	3M	*p*-value
**Anatomical parameters**			
**VT**	1.1165 ± 0.0226	1.0978 ± 0.0239	**< 0.001**
**SCP VD (%)**	48.06 ± 5.96	45.17 ± 4.11	**0.002**
**DCP VD (%)**	45.14 ± 6.43	48.32 ± 4.86	**0.029**
**FAZa (mm** ^**2**^ **)**	0.07 ± 0.10	0.08 ± 0.05	0.053
**FAZp (mm)**	1.04 ± 0.53	1.17 ± 0.38	0.140
**FAZ AI**	1.25 ± 0.20	1.19 ± 0.13	0.228
**RT (μm)**	359.93 ± 52.58	298.24 ± 26.87	**<0.001**
**Functional parameters**			
**MS (dB)**	22.27 ± 3.87	22.79 ± 2.63	0.451
**BCVA (logMAR)**	0.49 ± 0.38	0.22 ± 0.26	**<0.001**

Bold font indicates statistically significance (*p* < 0.05).

Abbreviations: iERM, idiopathic epiretinal membrane; VT, vessel tortuosity; VD, vessel density; SCP, superficial capillary plexus; DCP, deep capillary plexus; FAZ, foveal avascular zone; FAZa, FAZ area; FAZp, FAZ perimeter; FAZ AI, FAZ acircularity index; RT, retinal thickness; MS, mean sensitivity; BCVA, best-corrected visual acuity; logMAR, logarithm of the minimal angle of resolution.

The VT, SCP VD, and RT were divided into four quadrants in the macular area for further analysis at baseline, 1M, and 3M. At 1M, VT decreased significantly in the superior, nasal, and inferior quadrants (all *p* < 0.05). At 3M, VT also significantly decreased in the superior, nasal, and inferior quadrants (all *p* < 0.01). However, there was no significant improvement in the temporal quadrant at 1M or 3M (*p* = 0.785, *p* = 0.134, respectively). The SCP VD decreased significantly in four quadrants at 1M and 3M (all *p* < 0.05). Within 3 months, RT showed a steady decline for the entire macular area and all quadrants (all *p* < 0.01) (see Table 3; Figures 2A–C; Supplementary Table S1).

**TABLE 3:** Comparison of MS and anatomical parameters of different quadrants at baseline and 3 months (3M) after surgery in eyes with iERM.

Parameters	Baseline	3M	*P*_*1*_-value
**VT**			
Temporal	1.0957 ± 0.0219	1.1105 ± 0.1070	0.134
Superior	1.0962 ± 0.0214	1.0831 ± 0.0249	**<0.001**
Nasal	1.0993 ± 0.0153	1.0906 ± 0.0212	**0.001**
Inferior	1.0914 ± 0.0199	1.0830 ± 0.0236	**0.001**
*P*_*2*_-value	0.114	**<0.001**	
**SCP VD (%)**			
Temporal	44.02 ± 6.18	41.48 ± 4.75	**0.024**
Superior	47.72 ± 6.31	44.56 ± 5.36	**0.007**
Nasal	49.82 ± 7.26	47.11 ± 4.68	**0.013**
Inferior	47.57 ± 6.77	43.98 ± 4.42	**0.001**
*P*_*2*_-value	**<0.001**	**<0.001**	
**RT (μm)**			
Temporal	366.81 ± 76.93	286.33 ± 30.33	**<0.001**
Superior	387.06 ± 74.05	306.71 ± 29.51	**<0.001**
Nasal	388.79 ± 56.41	332.34 ± 28.68	**<0.001**
Inferior	357.69 ± 78.07	299.42 ± 28.97	**<0.001**
*P*_*2*_-value	0.076	**<0.001**	
**MS (dB)**			
Temporal	22.52 ± 4.05	22.46 ± 4.33	0.717
Superior	22.00 ± 4.18	23.61 ± 2.26	**0.010**
Nasal	22.27 ± 4.25	22.98 ± 2.46	0.488
Inferior	22.43 ± 3.72	22.72 ± 2.52	0.928
*P*_*2*_-value	0.860	0.400	

*P*_*1*_-value was obtained from the comparison of parameters at baseline and 3 months postoperatively.

*P*_*2*_-value was obtained from the comparison of parameters for the four quadrants.

Bold font indicates statistically significance (*p* < 0.05).

Abbreviations: iERM, idiopathic epiretinal membrane; VT, vessel tortuosity; VD, vessel density; SCP, superficial capillary plexus; RT, retinal thickness; MS, mean sensitivity.

At baseline, there were no significant differences in VT (*p* = 0.114) and RT (*p* = 0.139) across the four quadrants. The SCP VD of the temporal quadrant was the lowest among the four quadrants (*p* < 0.001). At 3M, there were significant differences in VT, SCP VD, and RT across the different quadrants (*p* < 0.001; *p* < 0.001; *p* < 0.001). Pairwise comparisons were made among the four quadrants. The VT of the temporal quadrant was more tortuous than that of the superior (*p* < 0.001) and inferior (*p* < 0.001) quadrants. There were no significant differences between the VTs of the temporal and nasal quadrants (*p* = 0.542). Compared to the other three quadrants, the SCP VD of the temporal quadrant was the lowest (superior *p* = 0.002; nasal *p* < 0.001; inferior *p* = 0.015). The RT of the temporal quadrant was thinner than that of the superior (*p* = 0.007) and nasal (*p* < 0.001) quadrants (see Table 3).

### Functional improvement

At baseline, the iERM group had worse MS and BCVA than those of the control group (MS 22.27 ± 3.87 dB vs. 26.18 ± 1.93 dB, *p* < 0.001; BCVA 0.49 ± 0.38 logMAR vs. 0.01 ± 0.04 logMAR, *p* < 0.001; see Table 1). During the 3-month follow-up after iERM surgery, the BCVA significantly improved to 0.22 ± 0.26 logMAR (*p* < 0.001). The MS of the macular region showed no difference after iERM surgery (*p* = 0.451), but there was a trend of increase (see Table 2; Figure 2D).

The MS of the superior quadrant significantly improved after iERM surgery (1M *p* = 0.043; 3M *p* = 0.010). In the three other quadrants, MS showed a trend of increase but with no significant difference (see Table 2; Figure 2D). Among the four quadrants, there was no significant difference in MS at baseline (*p* = 0.860) and 3M (*p* = 0.400) (see Table 3).

### Correlations between functional and anatomical parameters

We assessed the correlations between the functional and anatomical parameters of the macular region at 3M. There was a significant correlation between the BCVA and the MS at M3 (rs = −0.377, *p* = 0.015). The BCVA was associated with SCP VD, but not VT and RT at M3 (VT rs = −0.253, *p* = 0.111; SCP VD rs = −0.554, *p* < 0.001; RT rs = −0.076, *p* = 0.635). MS was associated with SCP VD and RT but not VT at M3 (VT rs = 0.077, *p* = 0.634; SCP VD rs = 0.511, *p* = 0.001; RT rs = 0.456, *p* = 0.003) (see Supplementary Table S2).

**FIGURE 3:**
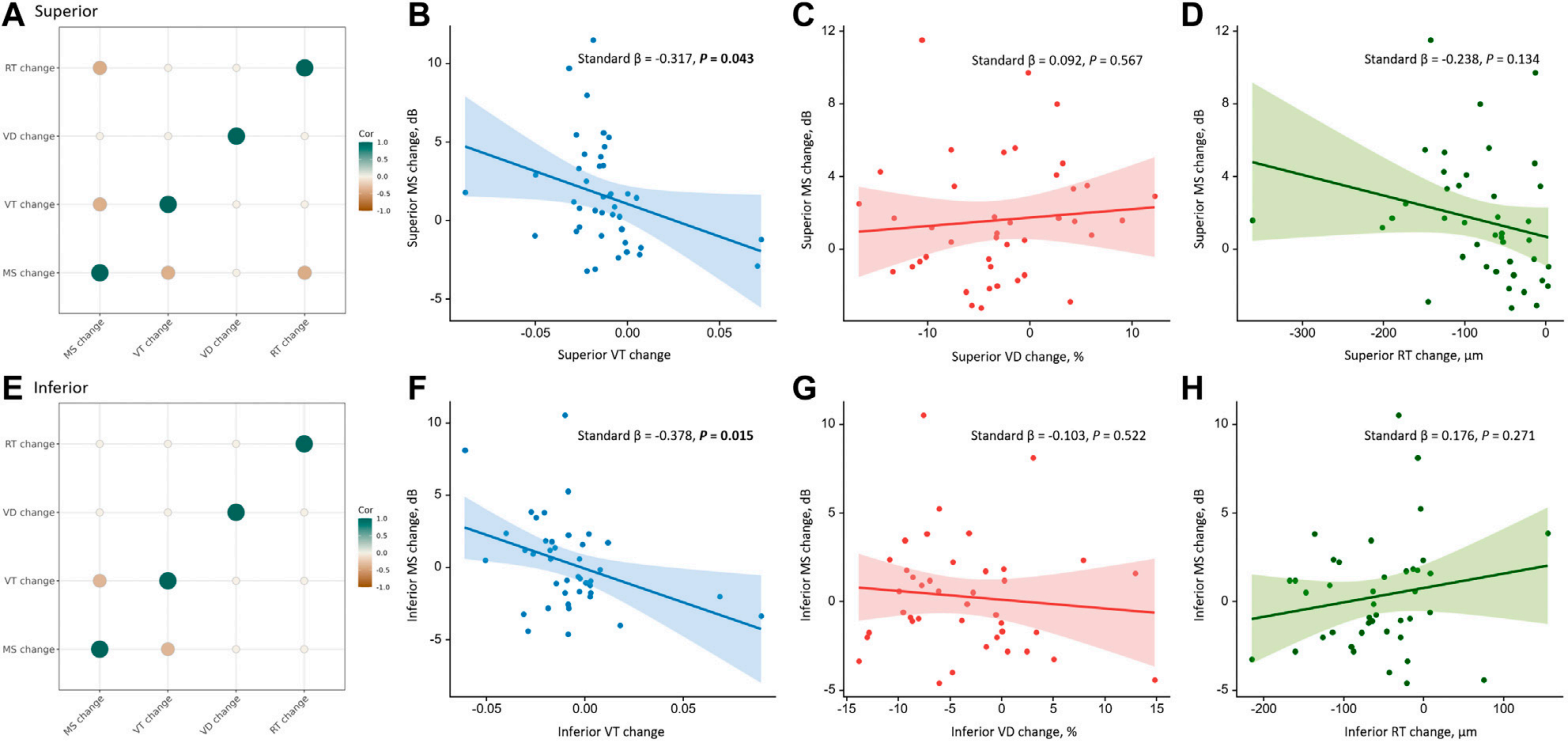
Correlations between the changes in anatomical parameters and the change in MS in the superior and inferior quadrants during the 3-month follow-up. The first column shows correlations among the changes in anatomical parameters and MS in the superior **(A)** and inferior **(E)** quadrants using Spearman’s Rho correlation. The color and size of the circle reflect the correlation tendency, degree, and significance. Only significant correlations with *p* < 0.05 are shown. The first row shows correlations of VT change **(B)**, VD change **(C)**, and RT change **(D)** with MS change in the superior quadrant using univariate linear regression. The second row shows correlations of VT change **(F)**, VD change **(G)**, and RT change **(H)** with MS change in the inferior quadrant using univariate linear regression. The solid line represents the regression line, and the dashed lines represent the 95% confidence interval of the regression line. Abbreviations: VT, vessel tortuosity; VD, vessel density; RT, retinal thickness; MS, mean sensitivity.

We further analyzed the correlations between the changes in MS and anatomical parameters in different quadrants during the 3-month follow-up after iERM surgery (see Figure 3). Using Spearman’s Rho correlation, a greater increase in MS was associated with greater reductions of VT and RT in the superior quadrant (VT, rs = −0.397, *p* = 0.010; RT, rs = −0.409, *p* = 0.008). In the inferior quadrant, a greater increase in MS was associated with a greater reduction of VT (rs = −0.349, *p* = 0.025). No correlation was found between the changes in VD and MS in the macular region and four quadrants (all *p* > 0.05) (see Table 4). Among the anatomical parameters, changes in VT were related to changes in VD in the temporal quadrant (rs = 0.350, *p* = 0.025; see Supplementary Figure S1E). No significant correlations were found between the changes in VT, RT, and VD in the macular, superior, nasal, and inferior quadrants (all *p* > 0.05; see Supplementary Figure S1).

**TABLE 4:** Correlation between the change in MS and anatomical parameters in different quadrants during the 3-month follow-up after iERM surgery using Spearman’s Rho correlation.

Quadrant	Anatomical parameters	rs	*p*-value
Macular	VT change	−0.130	0.418
	SCP VD change	−0.022	0.892
	RT change	−0.032	0.844
Temporal	VT change	0.009	0.955
	SCP VD change	0.085	0.596
	RT change	−0.007	0.963
Superior	VT change	−0.397	**0.010**
	SCP VD change	0.180	0.259
	RT change	−0.409	**0.008**
Nasal	VT change	0.151	0.347
	SCP VD change	−0.098	0.540
	RT change	−0.107	0.504
Inferior	VT change	−0.349	**0.025**
	SCP VD change	−0.114	0.478
	RT change	0.202	0.205

Bold font indicates statistically significance (*p* < 0.05).

Abbreviations: iERM, idiopathic epiretinal membrane; VT, vessel tortuosity; VD, vessel density; SCP, superficial capillary plexus; RT, retinal thickness; MS, mean sensitivity.

Univariate linear regression showed that an improvement in MS was associated with the reduction of VT in the superior (standard *β* = −0.317, *p* = 0.043) and inferior (standard *β* = −0.378, *p* = 0.015) quadrants, but not the macular region and temporal and nasal quadrants (all *p* > 0.05). Using multivariate linear regression, the improvement of MS was associated with the reduction of VT in the temporal (standard *β* = −0.322, *p* = 0.044), superior (standard *β* = −0.330, *p* = 0.034), and inferior (standard *β* = −0.480, *p* = 0.003) quadrants but not in the macular region and nasal quadrant (all *p* > 0.05). No correlation was found between the changes in SCP VD and RT with MS in the macular region and four quadrants (all *p* > 0.05) (see Table 5).

**TABLE 5:** Correlation between the change in MS and anatomical parameters in different quadrants during the 3-month follow-up after iERM surgery using multivariate linear regression.

Quadrant	Anatomical parameters	Standard *β*	*p*-value	Adjusted R-square
Macular	VT change	−0.020	0.905	−0.076
	SCP VD change	−0.063	0.707	
	RT change	−0.003	0.988	
Temporal	VT change	−0.322	**0.044**	0.052
	SCP VD change	0.171	0.281	
	RT change	−0.057	0.716	
Superior	VT change	−0.330	**0.034**	0.104
	SCP VD change	0.064	0.672	
	RT change	−0.255	0.098	
Nasal	VT change	−0.067	0.691	−0.063
	SCP VD change	−0.099	0.555	
	RT change	−0.067	0.684	
Inferior	VT change	−0.480	**0.003**	0.195
	SCP VD change	−0.274	0.076	
	RT change	0.261	0.080	

Bold font indicates statistically significance (*p* < 0.05).

Abbreviations: iERM, idiopathic epiretinal membrane; VT, vessel tortuosity; VD, vessel density; SCP, superficial capillary plexus; RT, retinal thickness; MS, mean sensitivity.

## Discussion

Our study assessed the impact of iERM surgery on retinal microvasculature. We showed that surgery may improve the structure of the retinal microcirculation over 3 months or even 1 month. We quantified and analyzed the vascular tortuosity of the retina in iERM using SD-OCTA. Previous studies have demonstrated that VT may be useful in differentiating the progression of DR (Klein et al., 2018; Alam et al., 2021). However, research examining VT in eyes with iERM has been limited. To our knowledge, this is the first study on vascular tortuosity and sensitivity in four quadrants before and after surgery for iERM. To obtain a more detailed and visually intuitive view, we focused on the microvascular characteristics of SCP. Additionally, the traction of ERM may alter the OCTA signal quality of DCP status, and it is necessary to prevent ERM-related projection artifacts.

Surgical treatment for ERM can release traction in the vitreoretinal interface, leading to vascular restoration and remodeling. Tangential and vertical macular tractions from ERM were considered the reason for vessel translocation and tortuosity (Yagi et al., 2012), which was supported by our observations that the VT in the eyes with iERM was higher than those in healthy eyes. With the release of tractional force generated from the ERM after removing preretinal tissue, the surgery facilitates the recovery of the main retinal vessels and capillaries to their original position. The VT significantly decreased after surgical treatment in this study, which may have contributed to the improvement of macular microcirculation. Consistent with the study by Miyazawa et al. (Miyazawa et al., 2022), more linearized vessels were found postoperatively.

Previous study demonstrated that FAZ would be enlarged and rounded after surgery (Hirata et al., 2019; Ersoz et al., 2021). This study obtained consistent results, and we suggested that the changes in FAZ were also manifestations of vascular restoration and remodeling after the release of traction. The formation of iERM may cause the centripetal contraction of retinal layers, accompanied by vascular displacement and contortion, which may contribute to the reduction of the FAZ area and the progressive deterioration of the standard circular shape of the FAZ. Our patients show more extensive AI than the controls, with smaller FAZa and FAZp. After ERM surgery, we still observed a decrease in AI yielding a more circular shape, although this was not statistically significant. Therefore, FAZ can reflect surgical efficacy at the end of peripheral blood circulation. Removing the retinal pucker can facilitate revascularization and resolve central vessel crowding and deformation.

**FIGURE 4:**
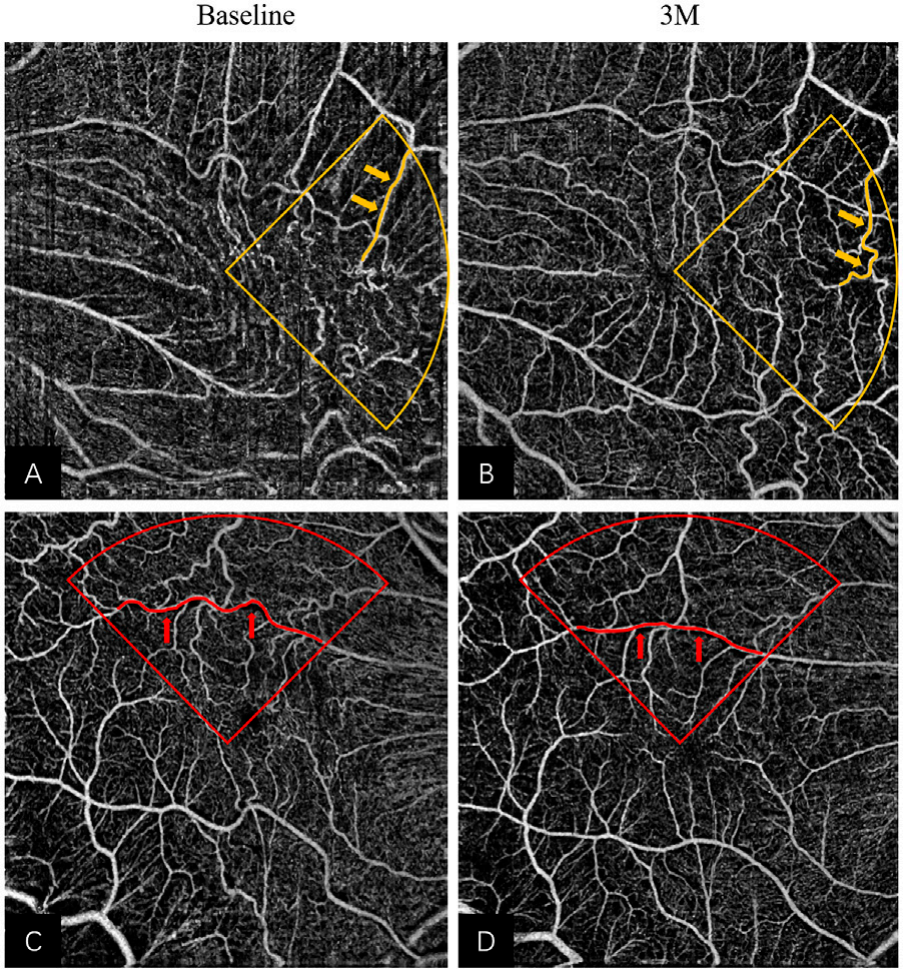
Remodeling of superficial retinal layer vessels in eyes underwent iERM surgery: **(A,B)** The pre- and post-operative OCTA images of a 65-year-old woman; more tortuous vessels (highlighted in yellow) were observed in the temporal quadrant at 3M in Figure 3B. **(C,D)** The pre- and post-operative OCTA images of a 57-year-old man; more linear vessels (highlighted in red) were observed in the superior quadrant at 3M in Figure 3D. Abbreviations: iERM, idiopathic epiretinal membrane; OCTA, optical coherence tomography angiography.

Notably, there were different anatomic remodeling patterns among the four quadrants in the macular area. We found that VT and MS showed no significant improvement postoperatively in the temporal quadrant (see Figures 4A, B). A reason may be that the nerve fiber layer was thinner, and the ganglion cells were fewer in the temporal retina than in other areas, which seemed more vulnerable to mechanical damage such as ILM peeling. Beyond that, the temporal blood vessels located at the end of retinal microcirculation may be more susceptible to the loss of structural support after membrane removal. As a result, the vessel network of the temporal region may be more disorganized, which may affect functional recovery. Conversely, the nasal macular area, localized at the proximal end to the optic disc, may be relatively stable and less influenced due to the high stability of the optic disc.

The most pronounced effect of VT was observed in the superior quadrant after treatment (see Figures 4C, D). The change in MS was associated with the change in VT in the superior and inferior quadrants using multivariate linear regression. However, the MS only improved significantly in the superior quadrant. Several studies have reported functional and structural asymmetries in the superior and inferior retina. In terms of functionality, the superior macular region may be more active. Some authors have found that the amplitudes of electroretinograms and the contrast sensitivity of the intermediate spatial frequencies are larger in the superior macular region than in the inferior macular region (Miyake et al., 1989; Silva et al., 2010), which suggests functional superiority of the upper retina. Secondly, there were anatomic differences between the superior and inferior retina. Earlier studies found ganglion cell and rod density were higher in the upper retina (Curcio and Allen, 1990; Curcio et al., 1990) and a recent study indicated that the blood flow in the superior retina was higher than that of the inferior retina (Tomita et al., 2020). Similar to these findings, our results showed appreciable MS improvement only in the superior quadrant. Taken together, the superior retina may show better vascular morphological restoration and blood flow reperfusion.

Our study presented a novel index for evaluating structural remodeling and blood flow perfusion. Additionally, we found the RT became thinner after surgery; however, the tortuosity of vessels in SCP reduced, which may also be the reason for the VD reduction in SCP. With the distorted and disorganized vessels tending to be normal, the resistance in the superficial vascular bed reduced, and the density decreased subsequently. Furthermore, the perfusion increased in DCP, suggesting the recovery of the anomalous tortuous capillaries dragged by the ERM and the improvement of blood flow in deep layers. Notably, the sensitivity improved after surgery, and MS was positively associated with RT and SCP VD 3 months after surgery. These may be because of retinal microstructural restoration and vascular perfusion after resolving the force exerted by the ERM, followed by the improvement of cell function. Several researchers have shown improvement of MS and BCVA in eyes with iERM postoperatively (Osada et al., 2020; Feng et al., 2021; Blautain et al., 2022). Consistent with these previous studies, visual acuity improved significantly and was associated with MS postoperatively in this study. As an example, Supplementary Figure S2 shows the images of a patient with iERM who had vessel remodeling in SCP and improved MS. However, the MS only showed a slight but non-statistically significant upward trend, which may be due to the limited sample size. Furthermore, anatomical features may recover relatively quickly, while visual function may demonstrate a chronic recovery course. This has already received our attention and will be further perfected and supplemented in subsequent studies.

There were several limitations of this study. First, the sample size was limited. Second, we only reported short-term outcomes, and long-term changes should be explored. Third, we focused on the vessels of the SCP to prevent or minimize artifacts, and future research on the microvascular characteristics of DCP is required.

## Conclusion

This study showed microvascular remodeling and perfusion recovery with a decrease in VT. BCVA improved significantly after iERM surgery and was associated with MS. In the superior macular quadrant, the reduction of VT was associated with the improvement of MS. Thus, VT may be a novel index for the morphology of the retinal microvasculature.

## Data Availability

The original contributions presented in the study are included in the article/Supplementary Material, further inquiries of source data can be directed to the corresponding authors.
