# Supplementary material for: Quantitative assessment of retinal microvascular remodeling in eyes that underwent idiopathic epiretinal membrane surgery
Source: Front Cell Dev Biol. 2023 Apr 20;11:1164529. doi: 10.3389/fcell.2023.1164529 (PMC10156972; doi:10.3389/fcell.2023.1164529)
Supplement: Supplementary file 1 [file Image2.pdf]

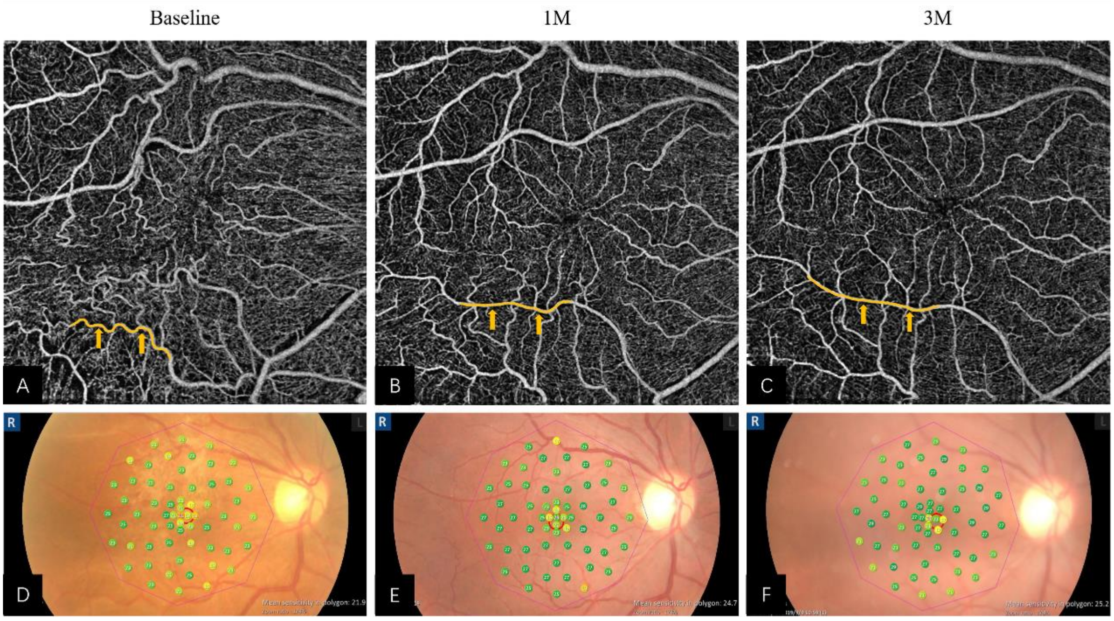

Figure S2. Remodeling of vessels in SCP and improvement of MS in a 57-year-old woman affected with iERM: Observed at baseline and 1 month (1M) and 3 months (3M) after vitreoretinal surgery. (A-C) More linear vessels (highlighted in yellow) in the SCP were observed after surgery; (D-E) Higher sensitivity was observed after surgery. Abbreviations: iERM, idiopathic epiretinal membrane; SCP, superficial capillary plexus; MS, mean sensitivity.
