# Supplementary material for: Quantitative assessment of retinal microvascular remodeling in eyes that underwent idiopathic epiretinal membrane surgery
Source: Front Cell Dev Biol. 2023 Apr 20;11:1164529. doi: 10.3389/fcell.2023.1164529 (PMC10156972; doi:10.3389/fcell.2023.1164529)
Supplement: Supplementary file 2 [file Table2.DOCX]

**Table S1. Comparison of MS and anatomical parameters of different quadrants at Baseline and 1-month (1M) after surgery in eyes with iERM**

| Parameters | Baseline | 1M | *P* value |
| --- | --- | --- | --- |
| **VT** |  |  |  |
| Macular | 1.1165 ± 0.0226 | 1.0982 ± 0.0109 | **< 0.001** |
| Temporal | 1.0957 ± 0.0219 | 1.0881 ± 0.0188 | **0.002** |
| Superior | 1.0962 ± 0.0214 | 1.0825 ± 0.0114 | **< 0.001** |
| Nasal | 1.0993 ± 0.0153 | 1.0926 ± 0.0168 | **0.014** |
| Inferior | 1.0914 ± 0.0199 | 1.0811 ± 0.0140 | **0.001** |
| **SCP VD (%)** |  |  |  |
| Macular | 48.06 ± 5.96 | 44.44 ± 4.77 | **< 0.001** |
| Temporal | 44.02 ± 6.18 | 40.34 ± 8.86 | **0.004** |
| Superior | 47.72 ± 6.31 | 42.75 ± 9.18 | **0.001** |
| Nasal | 49.82 ± 7.26 | 45.23 ± 9.89 | **< 0.001** |
| Inferior | 47.57 ± 6.77 | 42.33 ± 9.45 | **< 0.001** |
| **RT (μm)** |  |  |  |
| Macular | 359.93 ± 52.58 | 323.08 ± 39.13 | **< 0.001** |
| Temporal | 366.81 ± 76.93 | 307.95 ± 44.17 | **< 0.001** |
| Superior | 387.06 ± 74.05 | 326.77 ± 64.36 | **< 0.001** |
| Nasal | 388.79 ± 56.41 | 358.23 ± 40.98 | **0.002** |
| Inferior | 357.69 ± 78.07 | 329.05 ± 34.58 | **0.003** |
| **MS (dB)** |  |  |  |
| Macular | 22.27 ± 3.87 | 22.58 ± 3.02 | 0.992 |
| Temporal | 22.52 ± 4.05 | 22.13 ± 3.68 | 0.754 |
| Superior | 22.00 ± 4.18 | 23.28 ± 3.04 | 0.070 |
| Nasal | 22.27 ± 4.25 | 22.92 ± 2.82 | 0.855 |
| Inferior | 22.43 ± 3.72 | 22.19 ± 3.95 | 0.416 |

Abbreviations: iERM, idiopathic epiretinal membrane; VT, vessel tortuosity; VD, vessel density; SCP, superficial capillary plexus; RT, retinal thickness; MS, mean sensitivity.
