# Supplementary material for: Quantitative assessment of retinal microvascular remodeling in eyes that underwent idiopathic epiretinal membrane surgery
Source: Front Cell Dev Biol. 2023 Apr 20;11:1164529. doi: 10.3389/fcell.2023.1164529 (PMC10156972; doi:10.3389/fcell.2023.1164529)
Supplement: Supplementary file 3 [file Table3.DOCX]

**Table S2. Correlation between the functional and anatomical parameters in the macular region at 3-month after iERM surgery using Spearman’s Rho correlation**

| Functional parameters | Anatomical parameters | rs | *P* value |
| --- | --- | --- | --- |
| BCVA | VT | -0.253 | 0.111 |
|  | SCP VD | -0.554 | **< 0.001** |
|  | RT | -0.076 | 0.635 |
| MS | VT | 0.077 | 0.634 |
|  | SCP VD | 0.511 | **0.001** |
|  | RT | 0.456 | **0.003** |

Abbreviations: iERM, idiopathic epiretinal membrane; VT, vessel tortuosity; VD, vessel density; SCP, superficial capillary plexus; RT, retinal thickness; MS, mean sensitivity; BCVA, best-corrected visual acuity.
