# Supplementary material for: Quantitative assessment of retinal microvascular remodeling in eyes that underwent idiopathic epiretinal membrane surgery
Source: Front Cell Dev Biol. 2023 Apr 20;11:1164529. doi: 10.3389/fcell.2023.1164529 (PMC10156972; doi:10.3389/fcell.2023.1164529)
Supplement: Supplementary file 4 [file Image1.pdf]

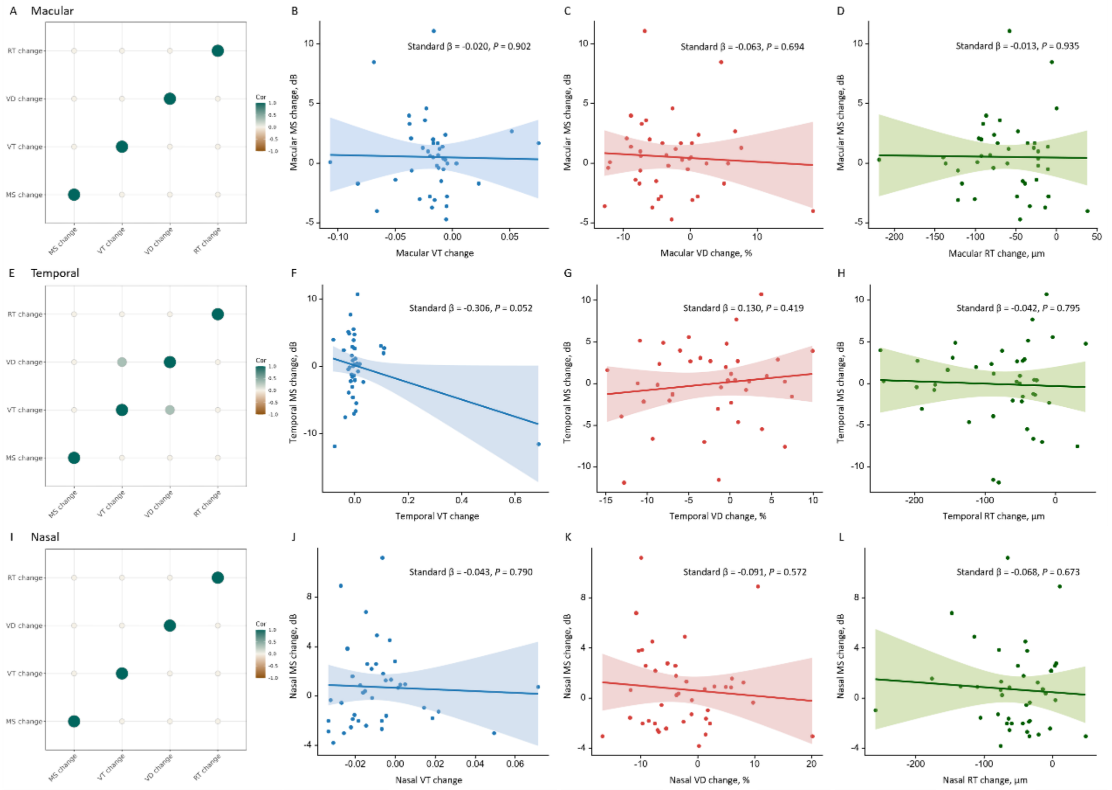

Figure S1. Correlations between the changes in anatomical parameters and the change in MS in the macular region and temporal, and nasal quadrants during the 3-month follow-up. The first column shows correlations among the changes of anatomical parameters and MS in the macular region (A) and temporal (E) and nasal (I) quadrants using Spearman's Rho correlation. The color and size of the circle reflect the correlation tendency, degree, and significance. Only significant correlations with $P < 0.05$ are shown. The first row shows correlations of VT change (B), VD change (C), and RT change (D) with MS change in the macular region using univariate linear regression. The second row shows correlations of VT change (F), VD change (G), and RT change (H) with MS change in the temporal quadrant using univariate linear regression. The third row shows correlations of VT change (J), VD change (K), and RT change (L) with MS change in the nasal quadrant using univariate linear regression. The solid line represents the regression line, and the dashed lines represent the 95% confidence interval of the regression line. Abbreviations: VT, vessel tortuosity; VD, vessel density; RT, retinal thickness; MS, mean sensitivity.
